# Enabling SDN in VANETs: What is the Impact on Security?

**DOI:** 10.3390/s16122077

**Published:** 2016-12-06

**Authors:** Antonio Di Maio, Maria Rita Palattella, Ridha Soua, Luca Lamorte, Xavier Vilajosana, Jesus Alonso-Zarate, Thomas Engel

**Affiliations:** 1 Interdisciplinary Centre for Security, Reliability and Trust (SnT), University of Luxembourg, 2721 Luxembourg, Luxembourg; ridha.soua@uni.lu (R.S.); luca.lamorte@uni.lu (L.L.); thomas.engel@uni.lu (T.E.); 2 Luxembourg Institute of Science and Technologies (LIST), 4362 Esch-sur-Alzette, Luxembourg; mariarita.palattella@list.lu; 3 Computer Science, Multimedia and Telecommunications Department, Universitat Oberta de Catalunya, 08018 Barcelona, Spain; xvilajosana@uoc.edu; 4 Centre Tecnològic de Telecomunicacions de Catalunya (CTTC), 08860 Castelldefels, Barcelona, Spain; jalonso@cttc.es

**Keywords:** smart cities, VANETs, Software Defined Networking, mobility, security, privacy

## Abstract

The demand for safe and secure journeys over roads and highways has been growing at a tremendous pace over recent decades. At the same time, the smart city paradigm has emerged to improve citizens’ quality of life by developing the *smart mobility* concept. Vehicular Ad hoc NETworks (VANETs) are widely recognized to be instrumental in realizing such concept, by enabling appealing safety and infotainment services. Such networks come with their own set of challenges, which range from managing high node mobility to securing data and user privacy. The Software Defined Networking (SDN) paradigm has been identified as a suitable solution for dealing with the dynamic network environment, the increased number of connected devices, and the heterogeneity of applications. While some preliminary investigations have been already conducted to check the applicability of the SDN paradigm to VANETs, and its presumed benefits for managing resources and mobility, it is still unclear what impact SDN will have on security and privacy. Security is a relevant issue in VANETs, because of the impact that threats can have on drivers’ behavior and quality of life. This paper opens a discussion on the security threats that future SDN-enabled VANETs will have to face, and investigates how SDN could be beneficial in building new countermeasures. The analysis is conducted in real use cases (smart parking, smart grid of electric vehicles, platooning, and emergency services), which are expected to be among the vehicular applications that will most benefit from introducing an SDN architecture.

## 1. Introduction

The integration of Information and Communication Technology (ICT) into a strategic approach to sustainability, citizen well-being, and economic development, has led to the concept of the smart city [1]. In other words, a city becomes “smart” when it takes advantage of ICT solutions to make better use of public resources, increase the quality of the services offered to its citizens, and reduce the operational costs of the public administration. In recent years, many cities have been installing sensors throughout the urban environment to capture data and use them to offer new services, such as smart collection of trashcans, management of traffic lights, assistance in finding for parking spots, or public transportation flow management, among many others. All of them, not only improve citizens’ life quality, but also bear operational saving and financial gains. For instance, smart parking solutions not only reduce driving time and pollution, but also raise the city’s administrative income from parking fees. In fact, by correlating the parking spot occupancy data with payment data, it is possible to discover infringements and apply sanctions accordingly. Similarly, smart sensors can detect when the trashcan should be emptied, consequently improving pick-up schedules and at the same time saving the city administration money.

Nowadays, particular interest in smart cities is focused on smart mobility, which includes enhancing traffic conditions, travel efficiency, vehicle safety, and drivers’/passengers’ comfort while on the road. Citizens want to connect to the Internet everywhere on the road, subscribe to a variety of services, and get real-time information about traffic and facilities (e.g., accident reports, road congestion, available parking spots, nearest ATM, gas stations, etc.). These services are mainly provided by vehicular applications which must be able to cope with the high mobility of the network environment and consequently with the unreliable connectivity, both in Vehicle-to-Vehicle (V2V) and Vehicle-to-Infrastructure (V2I) communication. Road Side Units (RSUs) are usually located only at a few critical intersection points with short radio communication ranges. Therefore, connectivity is intermittent: it is often broken and subsequently re-established in a different location [2].

Consequently, communication networks must be designed using a totally new approach, devising a new network paradigm that will be structured to minimize the impact of disconnections caused by vehicle mobility and improve reliability of communication in Vehicular Ad hoc NETworks (VANETs), fostering the development of smart mobility. The Software Defined Networking (SDN) paradigm [3] is a suitable candidate, and it is expected to result in a change from the way in which vehicular networks were classically operated. The first SDN architecture for VANETs was proposed by Ku et al. [4] in 2014 and later enhanced to add cloud and fog components [5,6].

Some aspects of the benefits offered by SDN to VANETs have been investigated in the literature [7,8,9,10], but never analyzed with respect to the impact that this network paradigm may have on security and privacy when applied in VANETs. In fact, security-related issues still represent a fundamental concern that could hinder SDN growth and development to its full potential [11]. At the same time, security is of prime importance in smart cities, where citizens will accept the adoption of the new technologies and services only if their privacy is not threatened. Security is also critical in VANETs because of the impact that attacks can have on people’s health and quality of life. Motivated by all these elements, in the present work we analyze how an SDN architecture could introduce new vulnerabilities in VANETs while also enabling novel security mechanisms to tackle well-known threats in VANETs.

The core contribution of this paper can thus be summarized as follows:

- A detailed survey of studies conducted by the research community for enabling SDN in VANETs is provided. While the applicability of SDN in vehicular networks has been discussed in several works, such a comprehensive overview with in-depth technical explanations is unprecedented.
- The awareness of the impact that SDN could have on security when applying such a paradigm in smart cities is raised for the first time.
- The introduction of a clear vision of the novel security countermeasures that SDN could build against some traditional attacks in VANETs, is totally novel and unprecedented.
- Finally, the applicability of the study in four concrete use cases shows the feasibility of the proposed solutions that are destined to be successfully implemented in future smart cities.

The present article is organized as follows: Section 2 provides an overview of related works that have proved the relevance of SDN for improving resource and mobility management in VANETs. Section 3 describes four use cases (smart parking, smart grid of electric vehicles, platooning, and emergency services) where the use of SDN may be beneficial, and where security threats may have relevant consequences. Section 4 focuses on the security concerns: we first identify the classical attacks in VANETs, and then the main security issues related to SDN. Section 5 describes how SDN could positively impact security in the considered use cases. Finally, Section 6 concludes the paper, summarizing main take-home messages.

## 2. SDN for Resource and Mobility Management

In this section, we first outline the main features of the SDN paradigm. Then, we analyze how this programmable approach applied to networking can be useful in easing the management of VANETs in future smart cities and providing Quality of Service (QoS) support for different types of service. By providing and overview of the few solutions already suggested in the literature, we highlight the advantages offered by SDN, which make it a good candidate for coping with smart city issues and especially for meeting the requirements of vehicular applications.

### 2.1. SDN: An Overview

The Software Defined Networking [3] paradigm introduces a centralized and programmable way of designing networks and was designed to overcome the drawbacks of traditional networks, such as manual configuration and maintenance of every single device in the network, high latency in path-recovery due to distributed approach, etc. SDN separates the data plane from the control plane, enhancing the programmability of the network by external applications. In an SDN-based network, the intelligence is centralized in a network controller, which determines how traffic flows will be forwarded within the network; while network devices (switches, routers) simply forward the packets, following the per-flow rules installed by the controller. By means of a Southbound Interface (SBI) and a Northbound Interface (NBI), the control plane can interact with the data plane and application plane respectively. By doing this, SDN simplifies network management. In detail, it provides highly dynamic, flexible and automated reconfiguration of the network, more efficient use of network resources, and makes troubleshooting [12] and debugging easier.

### 2.2. SDN in VANETs

**Figure 1:**
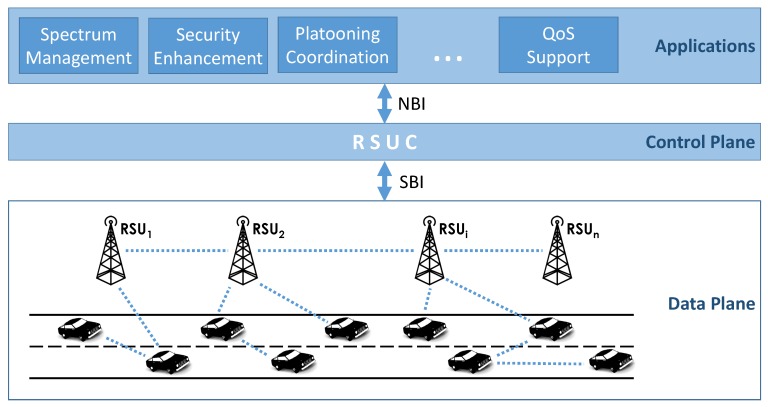
Reference SDN-enabled architecture. The RSU Controller (RSUC) converts the instructions received by the applications into rules that are executed by RSUs and vehicles.

Initially designed for wired networks, the SDN paradigm has also been widely recognized as an attractive and promising approach to improving wireless and mobile networks [13]. These can benefit from different aspects of the flexibility, programmability and centralized control offered by SDN, such as in wireless resource optimization (i.e., channel allocation, interference avoidance), packet routing in multi-hop multi-path scenarios, and efficient mobility and network heterogeneity management. Being just an approach, rather than a rigid architecture, it is feasible to apply SDN in a different context from the one it was initially designed for. In VANETs, for example, where the node mobility is high and the network topology changes frequently, the adoption of SDN is quite challenging. Several SDN-based architectures for VANETs have been proposed since 2014 [4,5,6]: all assume the presence of a central Road Side Unit Controller (RSUC) that can communicate with elements in the data plane and instruct vehicles and RSUs about the forwarding rules to apply and which resources to allocate for different traffic flows, related to distinct aspects (safety, efficiency, comfort, etc.). Figure 1 shows the reference SDN-enabled architecture for VANETs considered in this work. In a fully-centralized mode of operation, the architecture is prone to the well-known drawbacks of traditional wired SDN, e.g., the presence of single points of failure, loss of connectivity between RSUC and data plane, etc. Because of its increased reliability, a hybrid operating mode is preferable, in which the control of the network is shared between the RSUC and the local SDN agents that are installed in certain RSUs and vehicles. In this case, vehicles are equipped with two distinct interfaces for control channel communication, and for data channel exchanges [6]. While IEEE 802.11p (building on Wi-Fi) has been identified as the protocol for V2V and V2I communication in the data plane, the most suitable technology (among 4G, LTE, Wi-Fi, etc.) for the Southbound communication between the RSUC and the other elements in the network is still an open research question. The limited number of reserved channels versus the large number of applications to support, demands efficient spectrum management techniques. A SDN-enabled architecture can increase channel utilization, while keeping the collision probability low [7]. An initial contribution in this area is provided by Liu et al. [8], who have designed a cooperative data dissemination scheme for VANETs. An SDN agent, installed in the RSU, can deliver scheduling decisions to the vehicles in its coverage range, instructing them about which channel to tune to, and what data to transmit/receive. Moreover, a RSUC can estimate the frequency and duration of contacts between vehicles and RSUs, and thus the amount of data that they can exchange [9].

An SDN-enabled VANET has the advantage of dynamically adapting to topology changes by reconfiguring data forwarding rules in the network [5]. In this way, SDN minimizes service latency and improves user experience, while meeting the frequent and variable service demands of citizens and drivers. For instance, if a vehicle A is out of the coverage range of the RSU, but can get the content service from another neighbor vehicle B, which is currently within the RSU coverage range, the RSUC could allocate more resources to vehicle B to allow content delivery to vehicle A in an Information Centric Networking (ICN) fashion [14]. Similarly, an SDN controller can manage the number of data flows per RSU, making sure each flow is served according to the expected QoS [10].

Most of the infotainment applications in future VANETs will require the exchange large multimedia files. SDN has the potential to improve the management of content delivery [15] and in particular of content caching and forwarding [14,16]. Obviously, caching drastically reduces the delay for content discovery, retrieval and delivery in VANETs by providing multiple sources for the content. For instance, Soua et al. [14] propose using the RSUC and taking advantage of its global view of the network to identify the most popular content, according to a threshold, and the influential vehicles in which to cache such content. Alternatively, a push-and-pull approach for delivering content, based on content type, and on the number of users interested in it, namely Type-Based Content Distribution (TBCD), is proposed by Cao et al. [16].

SDN can also improve and reduce the number of broadcast messages exchanged in VANETs, with the consequent advantage of efficiently using network resources through collision reduction. Liu et al. use this capability in defining an SDN architecture for GeoBroadcast in VANETs [17]. Normally, every periodic warning message received by nearest RSU from the source vehicle is routed to the control center in the ITS (Intelligent Transport System), where it is redirected to every other RSU located in the geographical area for broadcasting. This produces a considerable overhead, and thus a waste of bandwidth. In [17], the first warning message received by the source RSU is sent to the SDN controller, which sets up the paths to the destination RSUs, and installs flow entries on RSUs and intermediates nodes. These paths will be followed by all the other periodic warning messages to broadcast. Therefore, controller overhead, message latency, and network bandwidth consumption are all reduced at the same time. Such a scheme can improve performance in many scenarios, both static (e.g., car accident) and dynamic (e.g., clear the way for an ambulance).

As highlighted in this section, the SDN paradigm can introduce several advantages when applied to VANETs, but we are still far from a seamless integration of SDN in the vehicular environment due to many challenges to be faced, most of them inherited from its mobile nature.

## 3. Use Cases in Future SDN-Based VANETs

Smart cities enhance the performance of urban services through tight integration of several sectors using ICT. Each sector can have its own requirements for internal and external communication over the network. Modern networks allow multiple tenants to share an infrastructure and offer the capability of allocating resources to each tenant according to communication requirements. However, these requirements can change in real time, depending on events occurring in the city; thus it may be necessary to dynamically scale the amount of network resources allocated to tenants. This section presents several use cases are presented, where SDN can be instrumental in managing the network infrastructure to meet the requirements of the applications.

### 3.1. Smart Parking

One of the most well-known smart city applications is the smart parking system, already available in several real deployments [18,19]. The main communicating infrastructure includes sensors for parking and traffic detection, embedded into the ground. The sensor nodes communicate parking space availability to a central server. A centralized control system stores and processes all the data gathered from sensors, and the resulting information and services are usually offered to citizens by means of mobile applications and city panels. The communication infrastructure design started in the early days with multi-hop mesh network technologies but failed to cover wide areas due to the complexity and the cost of the deployment of relays using city facilities and infrastructure such as lampposts or traffic lights [20]. Most of the current systems use Low Power Wide Area Network technologies such as these developed by Sigfox (Labège, France), Ingenu (San Diego, CA, USA), Weightless (Cambridge, UK), and LoRa (San Ramon, CA, USA) [21]. This communication model facilitates integration with cloud applications, as the messages are delivered at the transport layer by the service provider. The smart parking service enables advanced parking management in cities and facilitates the development of new applications, such as on-street parking reservation, effective management of loading and unloading areas, and prioritization of drivers with reduced mobility or disabilities. It also enables access to restricted areas without human intervention.

The addition of an SDN controller to the infrastructure can aid the dissemination of parking availability in a given area through V2I/V2V communications or using communications between peers, as achieved with the Floating Content approach [14,22,23,24]. Floating Content (FC) is an opportunistic communication strategy that allows messages to be disseminated in a determined geographical area in a distributed fashion. The aim of this approach is to control the probabilistic nature of the dissemination by providing models that ensure a desired level of content diffusion. By using P2P communications, the system experiences a reduction of inquiries sent to the central server and the need for information panels, thus cutting costs for the central administration. In detail, the envisaged network architecture includes an SDN controller co-located within an advanced LoRA gateway, which is a RSU equipped with multiple interfaces that allow communication via different V2X technologies (IEEE 802.11p, or LTE). When, for instance, a parking area is fully occupied, the controller can instruct the RSUs in its coverage range to broadcast this information. The installation of the forwarding rules can use cellular communication, while the data flow can be conveyed with IEEE 802.11p WAVE Short Messages (WSMs). To convey the information to vehicles that are currently distant, but which are approaching the parking area, the SDN controller can also activate an Anchor Zone (AZ), as defined in the Floating Content (FC) paradigm, and vehicles will subsequently exchange the data via wireless V2V communication.

Smart parking is prone to several security threats, and in particular to hardware attacks, which can compromise the physical devices (e.g., through radiated and magnetic interference) or their communication interfaces (through jammers). The addition of an SDN controller in the parking system can be particularly beneficial in detecting such attacks. Usually, monitoring applications can be characterized [19] and hence any behavioral change in the data traffic flows may be used to detect security threats. In particular, the SDN controller could perform anomaly detection based on traffic analysis (e.g., on packet headers, or packet size, and other statistics). Smart parking raises several issues concerning the privacy and confidentiality of citizens. Obviously, the guidance of citizens to a free parking slot relies on sharing their position information, which will be stored in a large central database managed by authorities. If a malicious actor compromises this database, confidential information about drivers and passengers may be exposed. Furthermore, in some cities, access to some parking spaces is reserved for residents of specific neighborhoods or for people with disabilities [25]. An attacker could redirect ineligible vehicles to these special parking spaces, causing disruption in the city. Again, in this case, the detection of unexpected trends in data traffic flow, can allow the SDN controller to identify potential attacks.

### 3.2. Smart Grid of Electric Vehicles

One of the main goals of smart cities is to improve energy efficiency and reduce greenhouse gases emissions, to delay the climate change (also called *climate departure* in [26]) that is predicted to take place in a few years. Beyond well-known services such as efficient street lighting and efficient scheduling of traffic lights, another step in this direction would be to encourage the introduction of Electric Vehicles (EVs). Although many researcher have been, and are being applied to investigate electrical power applications in the public transportation sector [27,28,29], the key to consistently reducing vehicle emissions is to apply it to vehicles in the private market. One of the main obstacles to this is the tremendous impact the change would have on both the electricity and transportation sectors. However, the beneficial effects are clear, and not just from the point of view of the environment [30].

EVs are considered to be the future active elements of smart grids, the technology that integrates information and power networks to efficiently distribute electrical energy. The original smart grid concept was devised to manage the distributed and intermittent production of electrical energy from renewable sources. In such a scheme, EVs will be able to dynamically improve the storage capacity of the power network in two ways: by absorbing unexpected generation peaks from renewable sources, and by injecting electricity back to the grid, acting as a short-term or emergency power supply [31]. EVs will be the mobile portion of the smart grid, which will be connected to the power supply network (static portion of the smart grid) through fixed-location facilities known as Electrical Vehicle Supply Equipment (EVSE). The combination of EVs and EVSEs is defined as Electric Vehicle Infrastructure (EVI), in which communications can take place via several technologies such as ZigBee (based on IEEE 802.15.4), Wi-Fi (IEEE 802.11), Power Line Communications (PLC) and others [32]. When an EV is connected to a EVSE, the two exchange information, as well as power, through the power cable. In particular, the IEC 61851 standard [33] describes a communication and signaling protocol between EV and EVSE, to exchange information about the State of Charge (SoC), electricity price, distance to the next EVSEs and so on. It is very likely that, in the near future, such channels will be used more intensively and extensively, carrying new types of messages like the electricity trading bids and other information used by the smart grid applications [32]. Additionally, in some specific applications such as the exchange of information about the location of charging stations on a road, EVs rely on V2V communications enabled by the IEEE 802.11p standard [34].

Increasing the freedom for communication across the EVI increases the number of possible security threats that can arise, for example, malware can be carried by compromised vehicles and infect EVSEs, which in turn will infect more EVs. This can obviously not only have a negative impact on service availability and security, but also on secondary aspects like the robustness of the market system, in which users might unduly benefit from services without paying (*free-riding*). On the EV side of EVI, malicious software can bring about minor issues, like the incorrect display of indicators such as fuel gauge and tachometer, up to more serious malfunctions in the safety system, such as taking control of the vehicle throttle and brakes. Furthermore, since EVs take part in V2V communications, the smart grid of EVs inherits the security vulnerabilities of VANETs. The combination of EVI with smart grids is intrinsically complex and prone to security issues at several levels as discussed in Section 5.2. The application of the SDN paradigm in EVI can be beneficial in addressing these issues: an SDN controller can provide reliable, robust and secure power system monitoring, and can suggest a smart charging schedule to EVs under given energy constraints. It can also enable security mechanisms such as isolating malicious or infected EVs and EVSEs, so hindering them from accessing EVI services.

**Figure 2:**
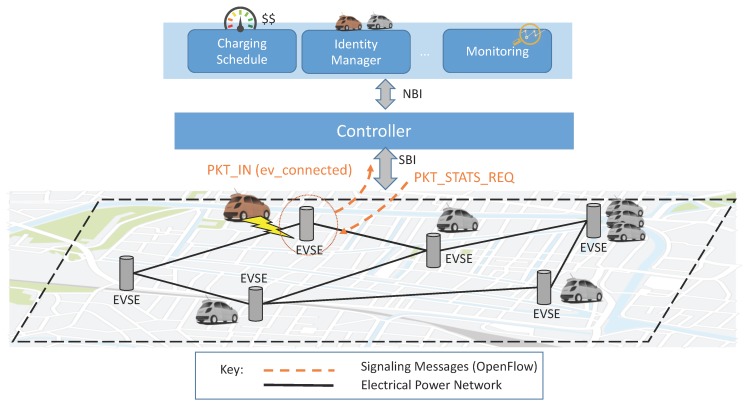
Generic architecture of an SDN-enabled EVI.

Figure 2 illustrates the SDN-based architecture that we propose for smart grids of EVs, where EVs and EVSEs represent the mobile and fixed elements respectively of the data plane. Since the smart grid is a wired network, we can imagine the OpenFlow protocol (with some adjustments) remaining the standard for the SBI communication used by the controller for collecting statistics (e.g., SoC, amount of energy remaining at EVSEs), and for imposing forwarding rules (i.e., the next EVSE at which to charge). Every time that an EV connects to an EVSE, a signaling message is sent back to the controller, which can keep track of the current topology and status of the network (e.g., level of charge of EVs). With the support of several applications, the controller can not only install forwarding rules, but also detect potential attacks implied by anomalous behaviors of data plane elements.

### 3.3. Platooning

**Figure 3:**
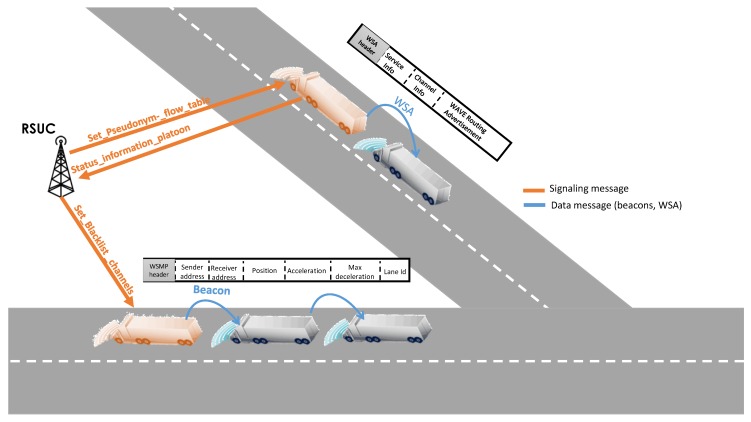
SDN-enabled platooning.

With the dramatic increase of drivers using roads and highways every day at peak times to reach their destination, there is an urgent need to control this large number of vehicles to ensure traffic fluidity and reduce air pollution. Moreover, safe driving on crowded highways requires attentive drivers, an accurate perception of the environment and critical decision making to react properly in emergency situations. These issues need to be tackled to provide smart mobility in smart cities. Platooning is one promising solution for addressing these problems [35]. Here, platooning means the ability to drive vehicles in controlled close formations called “platoons” at relatively small inter-vehicular distances. Vehicles behind the first in this formation are called followers, and receive downstream information from other vehicles in the platoon, as well as from a supervised vehicle known as the leading vehicle to adjust the distance between them and maintain the stability of the platoon [35]. Commonly, a platoon involves group of vehicles with common interests that benefit from having a trained, professional driver who has additional training for leading a platoon. Hence, platooning promises not only to provide smart mobility by also to enhance driving safety by reducing human involvement in the driving process. Indeed, a typical application of platooning is Cooperative Adaptive Cruise Control (CACC). In the classical Adaptive Cruise Control (ACC), vehicles rely exclusively on radar technology to sense an immediate (preceding or following) vehicle’s position and speed. This limited observation cannot efficiently handle inter-vehicle distance, resulting in poor highway capacities in the smart city. In contrast, CACC envisions that vehicles also receive information from the platoon leader via V2V communications. The purpose is to better optimize the inter-vehicular distance [35]. In particular, vehicles inside the platoon exchange periodic single-hop beacons (see Figure 3).

The leader vehicle plays a major role in platoon path planning, intra-platoon synchronization and collision avoidance. Thus, it is crucial to select the most appropriate entity to be the leader or to take control of the leader. One trivial solution is to have a human driver who orchestrates the platoon. However, this human leader is a part of a chain that is larger than the leading vehicle. This chain includes humans, vehicles and environment (roads, infrastructure, traffic signals, etc.). Consequently, this chain requires a central entity to manage the selection of an appropriate platoon leader and to establish its action profile, such as its acceleration and deceleration pattern, according to the city conditions. Since a smart city consists of complex transportation systems that are tightly connected, a central entity, namely the RSUC, might have coarse-grained control over multiple platoons and support each platoon leader, be responsible for intra-platoon issues, and wisely instruct follower vehicles [36].

In other words, the central controller can instruct the elements in the data plane (i.e., the platoon leader and follower vehicles) with appropriate rules for acceleration/deceleration, merging/splitting and changing lane according to real-time traffic conditions and unexpected events in the smart city. For instance, by using signaling messages exchanged over cellular (LTE) or wireless links (Wi-Fi or IEEE 802.11p), the RSUC can collect information on platoon status and abnormal vehicle behavior. On the dissemination side, the RSUC can instruct the leader on how to set specific parameters (acceleration, communication channels for data flow, scheduling policy of data messages, etc.) to ensure better network utilization and a safer journey. Such data messages convey information about distance and speed, for example, and are transmitted over IEEE 802.11p as beacon messages and WAVE Service Advertisement (WSA). The centralized orchestration provided by the RSUC allows the traveling distance over which the platoon stays intact to be maximized and therefore allows its lifetime to be extended. In addition, the presence of a centralized controller plays a major role in keeping the platoon secure and robust against cyber-attacks. For instance, the RSUC can support the platoon leader in a fast and efficient manner in detecting jamming and replay attacks as well as attacks targeting the management protocol. This latter is responsible for the splitting, merging, leaving and lane changing maneuvers. These threats are investigated in depth in Section 5.3.

### 3.4. Emergency Services

In large cities, the management of emergency situations that require the mobilization of the emergency services (e.g., firemen, medical services, police) becomes a difficult task when combined with traffic management systems. A quick reaction in such circumstances improves emergency management effectiveness and reduces its impact to the normal operation of the vehicular and transportation infrastructure. For example, emergency events occurring in the transport system may request the communication network to allocate additional communication channels or generally an increased amount of resources, in order to manage the event efficiently. A tight coordination of data network, traffic management systems and emergency services requires an adaptation of network data flows to raise the priority of actions that will affect the immediate handling of the emergency, such as quick traffic light actuation, deviation of traffic flows, and reservation of parking spots. More generally, the communication requirements between Emergency Services and traffic management systems in smart cities can range from best-effort to real-time depending on the type of information being exchanged and the urgency of the communication. Rather than statically over-provisioning resources to satisfy the possible needs of different types of communication, the SDN paradigm allows the flow of information between actors to be dynamically controlled in a proactive or reactive manner. This offers the ability to proactively provide a minimum amount of resources to these services and reactively scale according to changes in application needs.

SDN can help to disseminate emergency messages about an event that has occurred in the network by orchestrating Floating Content replication [14,22,23]. When an accident occurs, the vehicles involved, as well as witness vehicles, can start to disseminate information about the event using opportunistic communication. Specifically, the first vehicle that detects the anomaly (the seeder) creates an Anchor Zone (AZ) representing the geographical area where vehicles interested in the content are located [23,37]. When a vehicle in the AZ meets another vehicle that has a copy of the message, it receives it via V2V communication and stores it until it exits the AZ. In this context, the SDN controller can help the seeder vehicle to determine the best AZ parameters (shape, size) to optimize dissemination, according to global mobility parameters that are estimated regularly by the infrastructure.

**Figure 4:**
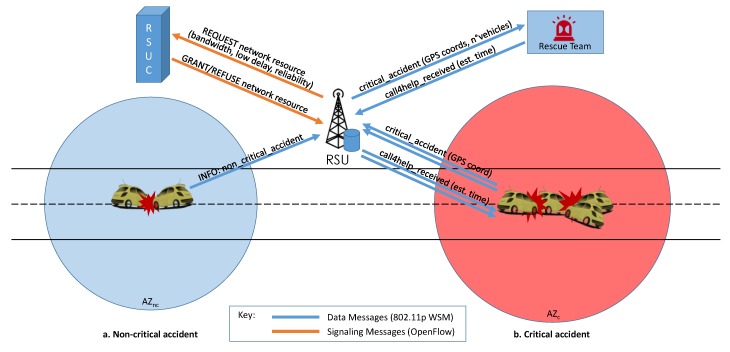
Emergency scenarios: (**a**) Non-critical accident: the vehicles start an AZ to disseminate local information about the moderate issue and optionally can send a Wave Short Message (WSM) notification to the RSU; (**b**) Critical accident: the vehicles involved create an AZ with a bigger radius than the non-critical case and notify the RSU about the entity of the accident, plus their location. In turn, the RSU sends OpenFlow messages to the RSUC to signal the need for more network resources, and waits for request approval or denial. At the same time, the RSU calculates the number of vehicles involved and sends an aggregated WSM request to the rescue service, which answers with the estimated arrival time that, in turn, will be forwarded to the vehicles concerned.

If the accident requires prompt action from the rescue service, for example when peoples’ lives are threatened, the SDN controller can rely on the infrastructure and ask RSUs to contact the first aid service via cellular networks, announce the event to surrounding vehicles, and broadcast IEEE 802.11p messages on the service channel as summarized in Figure 4.

The synergy between the centralized (infrastructure-based) and the distributed (FC-based) solutions can provide a quick local traffic redirection and a fast notification of the critical accident to the emergency vehicle, which will benefit of clear roads in the neighborhood of the event.

Furthermore, SDN can also help to mitigate some typical security breaches in the Emergency Service scenario, such as the Sink Hole Attack and the DDoS attack, by enforcing a trust scheme, as described in detail in Section 5.4.

## 4. Security of SDN and VANETs

The awareness that smart cities are exposed to several cybersecurity challenges is rapidly increasing, as shown by the creation of the global Securing Smart Cities initiative (http://securingsmartcities.org/), which aims to find workable solutions to make our cities cyber-safe. Security is of foremost importance in smart cities since business opportunities are strongly dependent on regulation concerning privacy and trust in the technology. To increase market demand for new emerging applications, citizens should be confident that data exchanges are secure, private, and that their personal information is kept confidential. In this section, we first review the classical security and privacy issues in VANETs. Then, in order to estimate the security threats that SDN could introduce when applied in the vehicular domain, we study the vulnerabilities of SDN from a security perspective.

### 4.1. Security Threats in VANETs

The consequences of a security breach can be critical or dangerous for drivers and passengers. Vehicular applications rely heavily on the messages exchanged, which impact drivers’ behavior. By substituting a message, or sending fake data, a malicious user can threaten security in several ways, for instance by generating traffic jams or, even worse, making the whole VANET unavailable (i.e., Denial of Service, DoS, attack), which can be dangerous if an emergency situation occurs. Thus, each vehicle should be able to establish the reliability of the received message, based for example, on the reputation of the sender vehicle.

To be cyber-safe, VANETs should meet the classical security requirements: Authenticity, Availability, Confidentiality, Data Integrity, and Non-Repudiation. The large scale of the network, the high mobility of the nodes, the uncertainty of their relative geographic positions, the intermittent connectivity between the nodes, and the unreliable channel conditions, make it difficult to satisfy these requisites [38]. There are several varieties of possible attacks for breaking security in VANETs. A generic classification of attacks is proposed in [39], categorizing them as: Bogus Information, DoS, Impersonation, Eavesdropping, Message Suspension, and Hardware Tampering. The literature classifies attacks mainly according to the specific requirement they compromise [40,41], or the layer of the protocol stack they impact [42]. For example, message tampering/suppression impacts data integrity, and if data is manipulated with the intention of erasing action traces and hampering driver identification, then it also impacts non-repudiation. From the protocol layer perspective, eavesdropping and jamming are attacks on the physical layer, while traffic analysis operates at the application layer, extracting private information from the data exchanged between vehicles (e.g., vehicle ID, location, etc.). Table 1, at the end of this section, summarizes some of the main attacks in VANETs.

Authentication represents the first step towards securing VANETs: all RSUs and vehicles in the network should register and get certificates from a Trusted Authority (TA). Before sending a message, each vehicle should first digitally sign the message. By checking the signature, the receiver can verify the integrity of the message, as well the identity of the sender. Despite these advantages, authentication poses a *privacy risk*, given that the TA will be aware at any time of each user’s specific location. An analysis of the security techniques that can secure VANETs while preserving privacy is presented in [39]. Several anonymous authentication schemes have been proposed to cope with this issue, and they can be grouped into three categories: group-signature-based schemes, pseudonymous authentication schemes, and hybrid schemes. In brief, to hide the vehicle identity, and avoid it being traced, the schemes use group signatures and pseudonymous authentication. While these increase the level of privacy, they may impact availability and safety, due to the high computational effort needed to verify the signature [41]. Trust schemes may be a solution to alleviate the costs and downsides of authentication, while providing a means of verifying sender reliability and data integrity.

Despite the numerous and different methods proposed so far in the literature, VANETs cannot yet be considered cyber-safe, and there is still a need to design new approaches that can preserve user privacy, while protecting VANETs from attacks.

### 4.2. Security Threats in SDN

The adaptation of SDN in smart cities will offer authorities the opportunity to run and upgrade different applications in a scalable manner, as they appear, without being concerned about network and hardware complexity. Despite the foreseen tangible benefits of SDN in improving resource allocation and network management (described in Section 2), SDN comes with its own vulnerabilities, inherited from its architecture, and main features: Centralized Controller, Abstraction and Programmability, and Flow-Based Forwarding.

Firstly, the controller represents a single point of failure, and the primary object of attack for malicious users. Among the different possible attacks, a typical one is *controller identity spoofing*: a malicious user plays the role of a fake controller, with the goal of manipulating the network [43]. This may have life-threatening consequences. For instance, a compromised SDN controller in a VANET can redirect drivers to hazardous areas or toward busy highways, which may result in acute traffic congestion and a dramatic increase in road casualties. Therefore, the SDN controller should be protected from DoS attacks and vulnerable open ports and protocols [43]. In real deployments, multiple controllers are implemented, each having its own security level. In this case, lack of orchestration between controllers could increase their vulnerability. Indeed, an attacker will select the less secure controllers to compromise flow tables [44]. The easy programmability of the network offered by SDN, coupled with the abstraction of flows and underlying resources, magnifies the vulnerability of SDN-bases systems to security breaches, malicious access and use. In fact, malicious software can be easily used to reprogram the entire network to exploit it for a harmful purpose. In VANETs, this kind of vulnerability allows the attacker to disseminate critical information without encryption instead of sending it encrypted. This forged behavior may undo the efforts made by authorities to efficiently disseminate critical information.

Finally, flow based-forwarding, the third key component of SDN, can also be a source of attacks. Similar to *route-poisoning* attacks, misbehaving nodes can inject bogus flows to saturate forwarding devices [45]. Since data plane nodes do not have sufficient intelligence, malicious flows cannot be detected. Subsequently, by sending faked flows, an attacker can exhaust the memory and cache of routers and switches, resulting in network dysfunction. For instance, in emergency situations, injecting malicious flows in the VANET will degrade the ability of vehicles to forward packets, since their memories are saturated. Consequently, safety messages will not be relayed to traffic authorities, possibly leading to fatal accidents.

However, by analyzing SDN’s main features from a different perspective, it can be revealed as beneficial and useful in implementing novel security mechanisms, which can be successful where other traditional mechanisms failed. In smart cities, citizens will be connected using a diverse set of technologies such as LTE, GPRS, UMTS, ZigBee, Bluetooth and GSM. Each independently implements a specific security policy without coordination. This can lead to potential policy conflict. For instance, backhaul protection in LTE networks uses IPSec, while in VANETs Public Key Infrastructure (PKI) is used to prevent malicious users from jeopardizing the network. Handling these myriad security mechanisms in a cooperative manner is not trivial. Here, the role of the controller is vital: with its global view of the network, it is able to deploy a huge set of security policies while avoiding overlap and conflict between them, and optimizing network resources. The global awareness of the controller drastically reduces the complexity of security policy deployment and optimizes overall network performance.

SDN’s abstraction from the underlying hardware resources can also be helpful in improving security. In fact, diverse security mechanisms, such as multi-access technologies, wire sniffers, firewalls, etc. [45], can be deployed without the burden of hardware complexity and compatibility. In SDN based systems, forwarding devices are simple and do not process security standards: these devices only accept instructions from the central controller. Hence, security administrators can programmatically configure/upgrade/remove security policies in these unintelligent devices according to the observed improper behavior without having to hand-code policies for many diverse devices scattered over the smart city.

Finally, SDN allows per-flow-based granular security management to be implemented. In SDN, all packets belonging to same flow are handled by the same service policy at the data layer. Therefore, if some flows are identified as suspicious, it is possible to efficiently label and isolate them; forwarding devices will not be burdened with handling packets coming from these labeled flows. Table 2 summarizes the security threats and opportunities offered by SDN, described in this section.

## 5. Improving VANETs Security Using SDN Paradigm

This section focuses on security benefits introduced by the application of SDN in VANETs. For each use case we describe in detail how SDN, with its programmability and monitoring features, can help in building new countermeasures against classical attacks in vehicular networks.

### 5.1. Smart Parking

Smart parking relies on the communication between intelligent sensors, deployed at each parking spot, and the gateway, using wireless technologies such as Zigbee, LoRa and Wi-Fi. These networks expose a large number of vulnerabilities and are subject to a wide range of attacks. We detail two types of attacks: jamming and eavesdropping, which target service availability and data confidentiality/privacy respectively.

**Figure 5:**
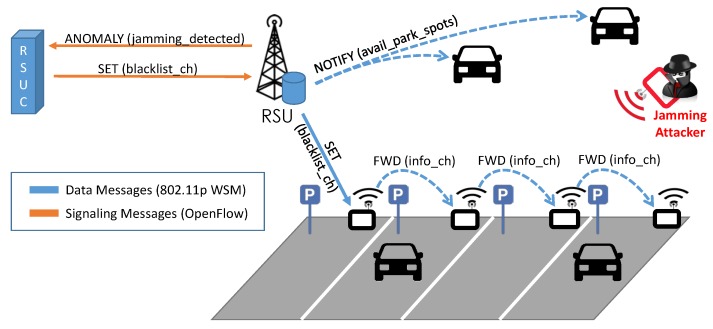
Jamming attack on a smart parking.

Given the broadcast nature of wireless communication, an outsider attacker can jam the network by using a transmitter more powerful than those embedded in sensors, as shown in Figure 5. This attack hinders the reception of sensed data by the WSN gateway, preventing information about parking spot availability from being transmitted to the RSU. Thus, the RSUC is not able to guide or schedule vehicles in the neighborhood currently asking for parking spots. The intelligence and the dynamic programmability of SDN can solve the problem of service disruption due to transmission jamming: the RSU gathers detailed information about the quality of channels used in the smart parking area and the report is then forwarded to the RSUC via specific signaling messages (jamming_detected). The RSUC builds a list of bad channels and asks the RSU to forward this list to sensors deployed in the parking area. Moreover, the RSUC can instruct sensors on how to accomplish channel hopping in order to mitigate heavy interference by providing a hopping schedule that the simple sensors can follow.

As stated in Section 3.1, one of the main security concerns in smart parking is ensuring drivers’ privacy, considering that the SDN controller acquires information about vehicles’ positions to build its global view of the network. A malicious vehicle can drive in the neighborhood of a parking area and gather, store and analyze the beacons of vehicles currently parked or looking for a parking spot. This attack is classically known as snooping (or eavesdropping) and aims to build information about the victim(s) from the sampled data. In particular, the attack can be directed to a specific victim, trying to reconstruct its habits or its driving path by analyzing its beacons, or it can be directed to the entire driver community, studying large-scale patterns to predict its behavior. The key to solving this problem is decoupling the ID from the vehicle, which can be achieved by using a pseudonym system [46] that assigns the user a temporary ID and switches it under certain conditions. The conditions required by this strategy are perfectly suited to a parking area: the ID can be randomly switched among a set of cars that wait in a small geographical area for some time. In this way, it is impossible to keep track of all the movements of a vehicle, eliminating the risk of single-targeted attacks.

**Figure 6:**
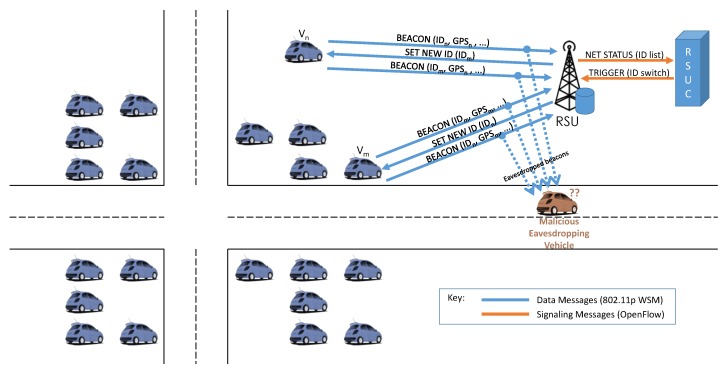
Eavesdropping/snooping attack in a smart parking area.

For example, Figure 6 presents a typical smart parking scenario in which an eavesdropping attack is being performed, and how the SDN controller counteracts. The vehicles $V_{n}$ and $V_{m}$ broadcast beacons with a certain frequency, which will be received by the RSU but also eavesdropped by the attacker. The intelligence in the RSU creates a list of the IDs in the area and sends it to the RSUC, which, according to its policies, can trigger an ID switch for a subset of vehicles. In the example, $V_{n}$ and $V_{m}$ exchange their IDs and, when they rebroadcast their beacons with the new ID, the attacker is unable to associate past and current information to the same entity anymore.

### 5.2. Smart Grid for Electric Vehicles

In the EVI, vehicles (mobile nodes) and the Smart Grid (fixed network) are frequently in contact, exchanging data and energy through specific interfaces that are particularly vulnerable to attackers. The safety of Vehicle to Grid (V2G) communications has been already studied in the literature [16]. However, some problems still need further investigation and more are likely to emerge given the huge penetration of EVs in the near future. By applying the SDN approach, the SDN controller can, with the support of monitoring and security applications, detect, isolate and mitigate attacks as soon as they appear in the network. One of the most common form of attack which can take place in the EVI is *Software Malware*. In fact, an EV could intentionally (or not) carry any sort of malicious software, which first infects the EVSE it is connected to, from there it may potentially spread the worm to other EVs or even worse, to the entire smart grid. The key to protecting the EVI from this type of attack is to strengthen the security of access points to the fixed infrastructure (EVSEs), at both protocol and physical level. Notably, the IEC 61851 protocol [33], which provides the specification for the physical layer signaling, can be prone to attacks that modify the normal sequence of handshaking signals before starting charging, and this can result in moderate to severe safety issues. In particular, malware affecting the physical layer can notify the EVSE about the support for a fast charge option on a vehicle that does not have it, leading the EVSE to provide the vehicle with an electrical power level that will not be tolerated by the internal charging component of its On Board Unit (OBU). The consequences of this malware attack can vary from shortening of battery life to battery explosion or vehicle fire. To mitigate this issue at the infrastructure level, SDN can help by detecting EVSEs that are acting suspiciously, for example supplying an incorrect amount of power for a certain type of vehicle or applying incorrect billing policies. Once detected, these suspicious EVSEs are isolated from the network until they are restored to a benign working state. Mitigating this type of attack can also be done at the network layer, as already thoroughly investigated in traditional wired OpenFlow based systems, using data flow analysis or exploiting other network features to infer suspicious vehicle behaviors.

**Figure 7:**
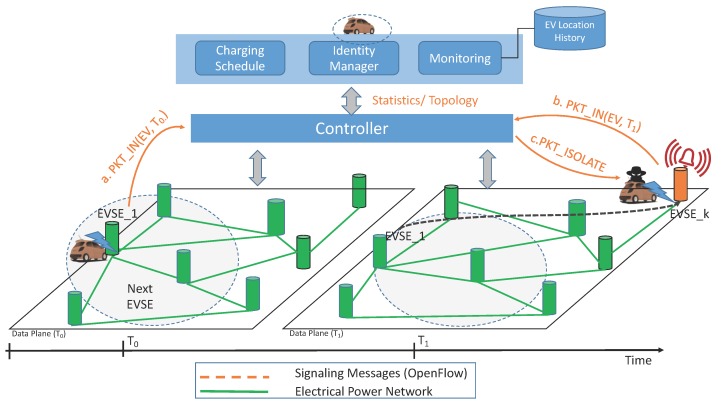
Masquerade attack on EVI.

EVs can also be subjected to Masquerade Attacks (i.e., identity theft), which impair the reliability of the whole system. The dynamic of the attack is illustrated in Figure 7. At time $T_{0}$, a certain EV demands to be charged by EVSE_1. Before commencing energy supply, the station informs the controller about the incoming flow through the message $Packet\_IN(EV,T_{0})$, which contains some information such as the vehicle identity. Collected flow information is processed by the applications connected to the controller through its NBI, helping the controller by providing information such as the list of the closest and available EVSEs, according to the spatial distance and the features of the vehicle battery, etc. As highlighted by the dotted area defined in the right part of Figure 7 (Data Plane $T_{1}$) , a vehicle that generally moves in a restricted area, is suddenly detected to be charging far away from the expected EVSE. According to the spatial locality principle, this vehicle is considered compromised and can be tagged as suspicious. In this situation the EV is possibly a victim of identity theft, and the controller should perform further inspections, for instance the user for identity confirmation or directly isolating the vehicle from the network. This is discovered by contacting the Data Plane at $T_{1}$, where the same EV appears at the station EVSE_k, which does not belong to the list of the EVSEs in the neighborhood of *EVSE_1*, provided to the EV at time $T_{0}$ by the Controller. With the PKT_Isolate command, the flow is promptly blocked and the EV cannot access the grid until further checks are carried out.

### 5.3. Platooning

Given the important role played by the platoon leader, one of the primary tasks of the central controller (RSUC) should be the careful selection of the trusted leader. To this end, Hu et al. [47] propose a recommendation scheme for follower vehicles to assist them in choosing the trusted leader. Basically, a server ranks the platoon leader candidates by establishing a trust and reputation system. The suitable candidates for the leader position are assessed after the server has collected feedback or reputation scores from following vehicles. To ensure that no malicious vehicle compromises the ranking procedure by injecting untruthful feedback, a filtering algorithm is used to exclude their feedback.

Besides privacy issues, several security attacks can be initiated in a CACC-enabled platoon, either by external (i.e., vehicles external to the platoon) or internal attackers (vehicles belonging to the platoon itself). Beacons messages normally used for intra-platoon communication, between the leader and the followers, are vulnerable to such malicious or misbehaving attackers. In the following section we describe how SDN could offer mechanisms for detecting and thwarting attacks from such malicious users. The analysis is conducted in the two distinct modes of platoon operation: the normal traveling and the platoon management maneuvers.

#### 5.3.1. Normal Traveling Mode

In this mode, the platoon is a stream of vehicles traveling on a straight highway. The platoon leader $P_{j}^{I}$ (where *I* denotes the Platoon id and *j* denotes the id of the vehicle inside the platoon) will trigger a slowing down action or a speeding up action to increase or decrease respectively the intra-platoon distance. To do this, the platoon leader relies on beaconing to exchange crucial parameters for a longitudinal control [48]. Position and acceleration are the most critical parameters in maintaining a safe gap between the stream of vehicles. An attacker can compromise safety by operating replay attacks and jamming attacks on beacons.

**Figure 8:**
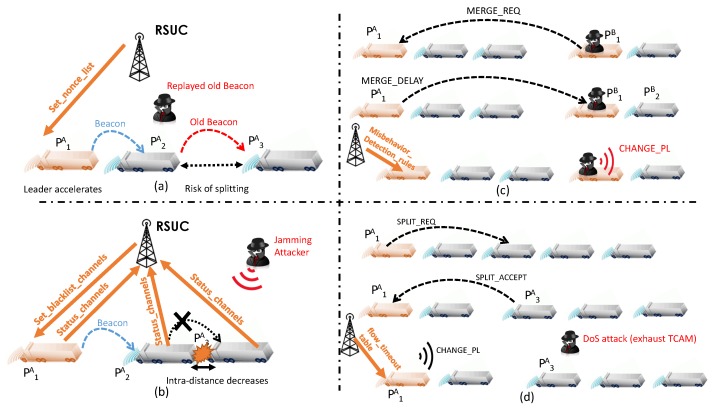
Different attacks on a platoon. (**a**) Replay Attack; (**b**) JammingAttack; (**c**) Insider Attack; (**d**) DoS Attack.

- Replay attack:
  The malicious user captures and replays a previously generated beacon at a later time or in other parts of the platoon. The replayed beacon includes expired information not relevant to the current situation of the platoon. For example, the platoon leader $P_{1}^{A}$ triggers an acceleration phase and sends a beacon to other followers to make them catch up. The adversary user periodically injects an old beacon with the old acceleration. Vehicles such as $P_{3}^{A}$ assume that the leader is driving at the normal speed and do not accelerate, potentially resulting to platoon splitting. In order to prevent replay attacks on platoons, the SDN paradigm can suggest two options. The first is to use a globally synchronized time for all vehicles. The central controller is a suitable candidate for providing a time reference making it pointless to inject old beacons into the platoon. The second option is based on the use of *nonce* numbers to uniquely identify each communication, preventing the malicious node from impersonating future communications inside the platoon. As shown in Figure 8a, these nonce values are generated by the RSUC and communicated to the trusted platoon leader via signaling messages (via the set_nonce_list command).
- Jamming attack:
  The attacker can be either a stationary or a moving jammer. Given the nature of the mobility of the platoon, it is rational to have a moving jammer that tracks the considered platoon and causes regular interference. The high level of interference coupled with the continuous aspect of this attacks makes it a thorny problem. As depicted in Figure 8b, the platoon leader $P_{1}^{A}$ broadcasts a beacon on the control channel (CCH), defined by the IEEE 802.11p standard, to instruct vehicles to slow down. $P_{2}^{A}$ re-broadcasts the beacon to the next follower vehicle. The malicious user jams the same channel and disrupts the correct reception of the beacon by $P_{3}^{A}$ and the remaining vehicles. Subsequently, vehicles inside the platoon will keep the same acceleration and hence can cause fatal collisions. SDN can mitigate the adverse consequences of jamming by dynamically selecting the channels on which beacons are sent: the central controller will provide the trusted leader with a channel blacklist that identifies channels the controller regards as “bad” (set_blacklist_channels command). This list is updated regularly, based on the general overview of the controller. Channels are removed from the blacklist if the badness metric is below a specific threshold. To realize this mode of operation, the RSUC can use the WSA message to announce to leaders the suitable communications channels to use for their further communications with followers. Then, to enable its follower vehicles to learn the blacklisted channels, the leader represents it as a bitmap and embeds it in its beacons. Beacons are then forwarded in the data plane using multi-hop communications. Thus, vehicles are able to re-broadcast beacons avoiding using channels experiencing jamming.

#### 5.3.2. Platoon Management Mode

During its journey, the platoon structure can undergo changes leading to it is becoming bigger or smaller stream of vehicles. The platoon management protocols handle three basic maneuvers: merge, split and lane change. Each maneuver is coordinated by exchanging a sequence of micro-commands [49]. In this section we analyze the possible attacks that can frustrate the correct operation of these maneuvers.

The platoon management protocol is based on a coordination approach. The leader initiates the desired maneuver and followers either obey or send requests to the leader. to him. In addition, the configuration data required for coordination (platoon depth, platoon size, platoon members, etc.) is stored by the leader and not divulged to followers. The latter dynamically join and leave the platoon. However, some attacks can affect the smoothness of these maneuvers. We highlight how an inside attacker can impede two main maneuvers (merge and splitting).

- Merge maneuver:
  Merge combines two successive platoons in the same lane to form one single platoon. As shown in Figure 8c, this maneuver is initiated by the the platoon leader of the rear platoon ($P_{1}^{B}$) when the platoon size is less than the predefined target platoon size. One way that $P_{1}^{B}$, the compromised vehicle, can frustrate this by extracting the platoon id of the leading platoon (via exchanged beacons) and sending a unicast MERGE_REQ to $P_{1}^{A}$, which is busy with another maneuver and replies with a unicast MERGE_DELAY message to delay the merging operation. However, instead of waiting for a specific time, the malicious attacker $P_{1}^{B}$ catches up with the front platoon and sends CHANGE_PL to all its followers to set $P_{1}^{A}$ as a leader. Consequently, the resulting platoon could exceed the predefined target size and followers of $P_{1}^{B}$ could obey to instructions not intended for them. $P_{1}^{B}$ is considered to be as a trusted insider vehicle that has been compromised by malicious software, complicating its detection. A novel SDN-based detection scheme can integrate both data-centric and behavioral mechanisms. The network can be used as a point of observation, which gives the central controller a holistic view of network activity. For instance, the central controller can use data-centric mechanisms to detect that the insider vehicle is transmitting incorrect data. Results are then reinforced using behavioral mechanisms, which check whether this insider node is behaving according to protocol management specifications. In the example in Figure 8c, a correctly-behaving $P_{1}^{B}$ should trigger another MERGE_REQ after receiving MERGE_DELAY from $P_{1}^{A}$.
- Splitting maneuver:
  A split maneuver divides a platoon into two successive ones in the same lane when, for example, platoon size exceeds the predefined maximum size. It can be initiated by a follower in order to become a free vehicle, or by a leader to make a space for a lane change. In Figure 8, the leader $P_{1}^{A}$ initiates the splitting maneuver by sending a unicast request SPLIT_REQ to $P_{3}^{A}$. Upon reception of a SPLIT_ACCEPT from $P_{3}^{A}$, $P_{1}^{A}$ sends a multicast message CHANGE_PL with its forwarding rules to its followers behind $P_{3}^{A}$ to announce the leader change. At this moment, a malicious user can mount a DoS by flooding the network with useless traffic to the vehicles that should receive the CHANGE_PL, so generating forwarding disruption in the data plane. A large amount of forged or faked traffic sent to vehicles implies that a large number of flow rules must be stored exhausting the TCAM (ternary content-addressable memory) in forwarding vehicles and preventing the installation of the rule for CHANGE_PL. Consequently, the instruction to change platoon leader is not received by follower vehicles. Meanwhile, $P_{1}^{A}$ reports split completion by sending SPLIT_DONE to $P_{3}^{A}$ so it can slow down. However, vehicles behind $P_{3}^{A}$ are not aware of the leader change and cannot follow the false leader instructions. In SDN-enbaled VANETs, a DoS attack can be mitigated by adjusting flow timeouts, making flow tables less prone to overflows. This adjustment is made by the trusted platoon leader based on information communicated by the Figure (flow_timeout_table) in Figure 8d). Another way of avoiding bogus flows is to establish access control lists. The SDN controller monitors all communication (beacons, WSA messages, etc.) and builds a comprehensive view of the network. The analysis of different messages allows the extraction of topological and forwarding information to build a holistic network graph with traffic flows. Incoming traffic is compared to a set of validated flow rules [43].

As highlighted in this section, SDN promises to dramatically simplify platoon management maneuvers and enable sophisticated countermeasures to overcome malicious users’ threats.

### 5.4. Emergency Services

Emergency services are also heavily impacted by the introduction of the SDN paradigm in VANETs. Emergency notification is a life-critical VANET service that has particularly demanding criteria for security, which SDN can strengthen. In an emergency, SDN can modify the amount of network resources allocated for that VANET portion and reroute the emergency flow towards the rescue service in an efficient and secure manner, coordinating data encryption and making sure that messages are delivered correctly. An attacker who compromises the SDN-enabled VANET, can reroute emergency requests preventing them from reaching needed rescue teams or alerting teams when they are not required so impairing the reliability and availability of the service.

**Figure 9:**
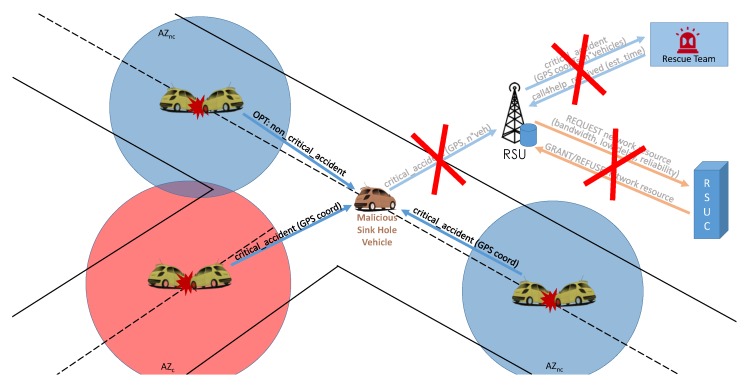
Example of sink hole attack on Emergency Services. The attacker receives and drops all the emergency requests from the vehicles in a certain area and this prevents the normal procedures of emergency forwarding and requests for special network resources. The red crosses over the faded arrows indicate the messages that would be exchanged in a regular scenario but, because of the attack, are not. The solution is to rely on SDN technology to elect only reputable intermediate nodes for V2V communication.

In traditional, distributed VANETs, a malicious vehicle can instruct a subset of nearby vehicles to send all their traffic through the attacker’s interface. SDN can provide an efficient strategy against this kind of attack, known in the literature as a Sink Hole attack. When a vehicle must act as a V2V intermediate node for communication, either towards infrastructure or another vehicle in the area, a trust-based authorization scheme, coordinated by the SDN controller, can be devised. In particular, the intermediate node must ask the SDN controller for authorization to act as a data relay, after the controller has computed its reputation in the opinion of the community. A generic vehicle that needs to send data to another peer must ask the SDN controller whether the recipient is dependable or not; this is determined by collecting feedback about it from the experience of community members. For example, a positive feedback can be provided from the members of the community when a certain vehicle forwards the traffic according to the rules of the protocol, while a negative feedback can be provided when the vehicle acts like a sink hole, gaining the right to act as a intermediate forwarding node but actually behaving like a Man-in-the-Middle. All the traffic, including emergency alerts will be routed through the malicious vehicle that, having full control of the information exchange, can decide to drop all the requests for help coming from an area, as shown in Figure 9.

If a malicious user takes control of a subset of vehicles that have been infected by malware (*zombie vehicles*), a particular case of Distributed Denial of Service (DDoS) applied to VANETs can occur. When the coordinating malicious vehicle (botmaster) triggers its infected vehicles subset (botnet), each infected node can start to originate fake emergency requests to overload the rescue team, thereby compromising the availability of the road safety service. In this case, SDN can identify the sources of the malicious traffic and then instruct the data plane (RSUs and vehicles) to drop the packets of those flows. In this way, the malicious network traffic is dropped directly at the routing device closest to the zombie vehicle. Furthermore, an SDN-based reputation management scheme for vehicles could be useful in identifying malicious users and exclude them from the network, using a shared consensus algorithm that is resilient to malicious collectives and does not present single points of failure [50].

Similarly, a malicious vehicle could notify a fake accident at a certain geographical location, so that the SDN controller reroutes the all other vehicles along a different path, allowing the malicious vehicle to benefit from a totally empty road. This is a typical example of a bogus information attack, and the SDN paradigm can effectively neutralize this threat using a collective consensus approach. After a vehicle signals an emergency in a certain area, the SDN controller collects related information from the other vehicles driving in that area. If the information collected from the other vehicles is inconsistent with that provided by the presumed malicious vehicle, the SDN controller sends a rule to its RSU to drop all the packets of that vehicles’ flows.

## 6. Conclusions

Connected vehicles in a smart city are evolving in a highly dynamic and complex environment that shapes driver decisions in critical situations. Specifically, Vehicular Ad hoc NETworks (VANETs) are widely accepted as a cornerstone for enabling safety, traffic and infotainment applications for drivers, passengers, and pedestrians in the smart city. Indeed, these highly-mobile networks are expected to contribute to road safety by providing pertinent information to drivers on potential threats within their surroundings. However, a VANET is not a benign environment, owing to its vast operating area and the underlying technologies used to deliver critical information. The wireless transmission of data using V2I and V2V communications makes it easily and constantly accessible to malicious users. Undoubtedly, in smart cities, security is fundamental to ensure a high level of availability and the integrity of services vital to citizens. While the complexity of smart cities is steadily increasing and evolving, traditional security measures and network management tools cannot practically cope with the explosively growing number of vehicles and the plethora of forwarding rules updates. To counter this thorny issue, Software Defined Networking (SDN) is proposed as an efficient paradigm that offers agility and flexibility to deal with the surge of vehicles and accommodate heterogeneous running applications. The first part of our paper provided an overview of SDN and the underlying architectures in VANETs, giving a special attention to new emerging applications in VANETs such as smart parking, platooning and electric vehicles. Our purpose was to investigate how these new applications can be orchestrated and managed by SDN. However, the new way of operating communication networks within SDN poses security and privacy challenges that must be solved so smart cities can be successful and accepted by citizens. To this end, we provided a comprehensive review of threat vectors in both VANETs and SDNs. For instance, platoon leader, electric vehicles and electric vehicle infrastructure could be the target of malicious users intent on wrecking vital services in a smart city. This investigation was particularly useful in understanding how SDN can overcome the weaknesses and vulnerabilities of current VANET architectures. Starting from this point, we provided some insights into the primary improvements required by SDN in the targeted use cases of VANETs. The intelligence of the SDN controller can detect improper behavior of electric vehicles or platoon members. Consequently, it can isolate these malicious nodes and reduce the resources allocated to them. Furthermore, the programmability of SDN allows security policies to be upgraded as the smart city evolves.

To the best of our knowledge, our paper is the first study that tackles the issues and new countermeasures introduced by SDN in SDN-enabled VANETs. However, there is a long path ahead of us before we can ensure a benign VANET environment where drivers and passengers can make their journeys without being compromised. We urge governments, standardization bodies and research institutions, along with car manufacturers to plan at all levels for making SDN a future security solution in our smart cities.

## Figures and Tables

**Table 1:** Non-exhaustive list of attacks in VANETs, classified by affected TCP/IP Layer, from 1 (PHY) to 5 (APP), and threatened Security Requirement (Security Req.): Authenticity (A), Availability (B), Confidentiality (C), Data Integrity (D), and Non-Repudiation (E).

Attack	Description	Security Req.					Affected Layer				
		A	B	C	D	E	1	2	3	4	5
Brute Force	The attacker tries all the possible key combinations to access a restricted entity or decrypt a piece of information	√		√							√
GPS Spoofing	The attacker broadcasts a forged GPS signal that overtops the legitimate one, so that vehicles get wrong position data	√	√				√				
Illusion Attack	The attacker spreads incorrect information on road conditions, influencing the route of nearby vehicles	√	√								√
Bogus Information Attack	Like Illusion Attacks, but here the attacker sends fictitious messages about road conditions for its personal benefit	√	√								√
DoS Attack	A huge amount of useless traffic is sent to the victim, to hamper its responsiveness to legitimate user requests		√						√	√	√
DDos Attack	As DoS, but the attacker controls a set of so-called zombie nodes that perform the attack in a distributed fashion		√						√	√	√
Jamming	A strong interference signal spoils the wireless traffic, so that no communication can happen on a specific channel		√		√		√				
Malware Attack	An attacker inject the VANET with a malicious piece of software that replicates itself via V2V communications		√	√	√						√
Masquerade Attack	The attacker steals the trusted identity of a reputable node and sends (possibly modified) messages on its behalf	√			√			√	√	√	√
Replay Attack	The attacker sniffs a message that it reuses as is, for authenticated access to a restricted network realm	√				√					√
Repudiation	The attacker denies being the sender of a message, causing retransmissions and therefore congestion		√			√					√
Sink Hole Attack	The attacker vehicle instructs a VANET portion to route all traffic to it, acting like a malicious gateway		√	√	√			√	√		√
Snooping	The attacker eavesdrops the network traffic at a certain layer without modifying it, in order to extract information			√			√	√	√	√	√
Man-in-the-Middle	Adds to the Snooping Attack the possibility of altering the message content and sending it to the original recipient	√		√	√	√	√	√	√	√	√
Spamming	Many messages are sent through the VANET to vehicles that are not interested in their content, typically ads		√								√
Sybil Attack	The attacker creates multiple fictitious identities to gain reputation and power in a trust management scheme	√									√
Timing Attack	The reliability of the infrastructure is compromised by delaying the forwarding of time-critical messages		√						√		√

**Table 2:** SDN: Security vulnerabilities and opportunities.

SDN Features	Security	
	Advantages	Threats
Centralized Controller	Avoid conflicts among security policies	Single point of failure
Abstraction and Programmability	Support for diverse security policies	Open to software vulnerabilities
Flow-based Forwarding	Isolate and label suspicious flows	Flow-poisoning attack

## References

[1] Pike Research (2016). Smart Technologies and Infrastructure for Energy, Water, Mobility, Buildings, and Government: Global Market Analysis and Forecasts. Online Report. http://www.navigantresearch.com/research/smart-cities.

[2] Cunha F., Villas L., Boukerche A., Maia G., Viana A., Mini R.A.F., Loureiro A.A.F. (2016). Data communication in VANETs: Protocols, applications and challenges. Ad Hoc Netw..

[3] Kreutz D., Ramos F.M.V., Esteves Verissimo P., Esteve Rothenberg C., Azodolmolky S., Uhlig S. (2015). Software-defined networking: A comprehensive survey. Proc. IEEE.

[4] Ku I., Lu Y., Gerla M., Ongaro F., Gomes R.L., Cerqueira E. Towards software-defined VANET: Architecture and services. Proceedings of the IEEE 2014 13th Annual Mediterranean Ad Hoc Networking Workshop (MED-HOC-NET).

[5] Salahuddin M.A., Al-Fuqaha A., Guizani M. (2015). Software-defined networking for RSU clouds in support of the internet of vehicles. IEEE Internet Things J..

[6] Truong N.B., Lee G.M., Ghamri-Doudane Y. Software defined networking-based vehicular adhoc network with fog computing. Proceedings of the 2015 IFIP/IEEE International Symposium on Integrated Network Management (IM).

[7] Wang W., Chen Y., Zhang Q., Jiang T. (2016). A software-defined wireless networking enabled spectrum management architecture. IEEE Commun. Mag..

[8] Liu K., Ng J.K.Y., Lee V., Son S.H., Stojmenovic I. (2015). Cooperative Data Scheduling in Hybrid Vehicular Ad Hoc Networks: VANET as a Software Defined Network. IEEE/ACM Trans. Netw..

[9] Xiao X., Kui X. (2015). The characterizes of communication contacts between vehicles and intersections for software-defined vehicular networks. Mob. Netw. Appl..

[10] Bozkaya E., Canberk B. QoE-based flow management in software defined vehicular networks. Proceedings of the 2015 IEEE Globecom Workshops (GC Wkshps).

[11] Ahmad I., Namal S., Ylianttila M., Gurtov A. (2015). Security in Software Defined Networks: A Survey. IEEE Commun. Surv. Tutor..

[12] Gheorghe G., Avanesov T., Palattella M.R., Engel T., Popoviciu C. SDN-RADAR: Network troubleshooting combining user experience and SDN capabilities. Proceedings of the 2015 1st IEEE Conference on Network Softwarization (NetSoft).

[13] Yang M., Li Y., Jin D., Zeng L., Wu X., Vasilakos A.V. (2014). Software-defined and virtualized future mobile and wireless networks: A survey. Mob. Netw. Appl..

[14] Soua R., Kalogeiton E., Manzo G., Duarte J.M., Palattella M.R., Maio A.D., Braun T., Engel T., Villas L.A., Rizzo G.A. SDN coordination for CCN and FC content dissemination in VANETs. Proceedings of the 8th International Conference on Ad Hoc Networks (ADHOCNETS).

[15] Broadbent M., King D., Baildon S., Georgalas N., Race N. OpenCache: A software-defined content caching platform. Proceedings of the 2015 1st IEEE Conference on Network Softwarization (NetSoft).

[16] Cao Y., Guo J., Wu Y. SDN enabled content distribution in vehicular networks. Proceedings of the 2014 Fourth International Conference on Innovative Computing Technology (INTECH).

[17] Liu Y.C., Chen C., Chakraborty S. A Software Defined Network Architecture for GeoBroadcast in VANETs. Proceedings of the 2015 IEEE International Conference on Communications (ICC).

[18] Worldsensing (2015). FasrPrk. Smart Parking System. http://www.worldsensing.com.

[19] Martinez B., Vilajosana X., Vilajosana I., Dohler M. (2015). Lean sensing: Exploiting contextual information for most energy-efficient sensing. IEEE Trans. Ind. Inform..

[20] Palattella M.R., Dohler M., Grieco A., Rizzo G., Torsner J., Engel T., Ladid L. (2016). Internet of things in the 5G era: Enablers, architecture, and business models. IEEE J. Sel. Areas Commun..

[21] Centenaro M., Vangelista L., Zanella A., Zorzi M. (2015). Long-range communications in unlicensed bands: The rising stars in the IoT and smart city scenarios. IEEE Wirel. Commun..

[22] Ali S., Rizzo G., Marsan M.A., Mancuso V., Vinh C.P., Alagar V., Vassev E., Khare A. (2014). Impact of Mobility on the Performance of Context-Aware Applications Using Floating Content. Context-Aware Systems and Applications, Proceedings of the Second International Conference (ICCASA 2013), Phu Quoc Island, Vietnam, 25–26 November 2013.

[23] Ali S., Rizzo G., Mancuso V., Marsan M.A. Persistence and availability of floating content in a campus environment. Proceedings of the 2015 IEEE Conference on Computer Communications (INFOCOM).

[24] Di Maio A., Soua R., Palattella M.R., Engel T., Rizzo G. A centralized approach for setting floating content parameters in VANETs. Proceedings of the 2017 IEEE Consumer Communications & Networking Conference (CCNC).

[25] Chatzigiannakis I., Vitaletti A., Pyrgelis A. (2016). A privacy-preserving smart parking system using an IoT elliptic curve based security platform. Comput. Commun..

[26] Mora C., Frazier A.G., Longman R.J., Dacks R.S., Walton M.M., Tong E.J., Sanchez J.J., Kaiser L.R., Stender Y.O., Anderson J.M. (2013). The projected timing of climate departure from recent variability. Nature.

[27] Jang Y.J., Ko Y.D. System architecture and mathematical model of public transportation system utilizing wireless charging electric vehicles. Proceedings of the 2012 15th International IEEE Conference on Intelligent Transportation Systems.

[28] Lam A.Y.S., Leung Y.W., Chu X. (2014). Electric vehicle charging station placement: Formulation, complexity, and solutions. IEEE Trans. Smart Grid.

[29] Wang X., Yuen C., Hassan N.U., An N., Wu W. (2016). Electric vehicle charging station placement for urban public bus systems. IEEE Trans. Intell. Transp. Syst..

[30] Shuai W., Maille P., Pelov A. (2016). Charging electric vehicles in the smart city: A survey of economy-driven approaches. IEEE Trans. Intell. Transp. Syst..

[31] Naik M.B., Ranjan A., Gunawat V., Kumar P., Majhi S. A brilliant public transportation system linked with electric vehicles in coordination with the grid. Proceedings of the 2014 Annual IEEE India Conference (INDICON).

[32] Mousavian S., Erol-kantarci M., Ortmeyer T. Cyber Attack Protection for a Resilient Electric Vehicle Infrastructure. Proceedings of the 2015 IEEE GLOBECOM Workshops.

[33] Webstore International Electrotechnical Commission IEC 61851-1:2010. Electric Vehicle Conductive Charging System—Part 1: General Requirements. https://webstore.iec.ch/publication/6029.

[34] Al-Anbagi I., Mouftah H.T. (2016). WAVE 4 V2G: Wireless access in vehicular environments for vehicle-to-grid applications. Veh. Commun..

[35] Jia D., Lu K., Wang J., Zhang X., Shen X. (2016). A survey on platoon-based vehicular cyber-physical systems. IEEE Commun. Surv. Tutor..

[36] Mc Goldrick C., Rabsatt V., Gerla M. (2015). Independent active ageing-the role of 5G and autonomous vehicles. E-Letter.

[37] Hyytiä E., Virtamo J., Lassila P., Kangasharju J., Ott J. When does content float? Characterizing availability of anchored information in opportunistic content sharing. Proceedings of the 2011 Proceedings IEEE INFOCOM.

[38] Engoulou R.G., Bellache M., Pierre S., Quintero A. (2014). VANET security surveys. Comput. Commun..

[39] Qu F., Wu Z., Wang F.Y., Cho W. (2015). A security and privacy review of VANETs. IEEE Trans. Intell. Transp. Syst..

[40] Mejri M.N., Ben-Othman J., Hamdi M. (2014). Survey on VANET security challenges and possible cryptographic solutions. Veh. Commun..

[41] Azees M., Vijayakumar P., Deborah L.J. (2016). Comprehensive survey on security services in vehicular ad-hoc networks. IET Intell. Transp. Syst..

[42] Mokhtar B., Azab M. (2015). Survey on security issues in vehicular ad hoc networks. Alex. Eng. J..

[43] Akhunzada A., Ahmed E., Gani A., Khan M.K., Imran M., Guizani S. (2015). Securing software defined networks: Taxonomy, requirements, and open issues. IEEE Commun. Mag..

[44] Scott-Hayward S., O’Callaghan G., Sezer S. SDN security: A survey. Proceedings of the 2013 IEEE SDN for Future Networks and Services (SDN4FNS).

[45] Liyanage M., Abro A.B., Ylianttila M., Gurtov A. (2016). Opportunities and challenges of software-defined mobile networks in network security. IEEE Secur. Priv..

[46] Wang J., Zhang Y., Wang Y., Gu X. (2016). RPRep: A robust and privacy-preserving reputation management scheme for pseudonym-enabled VANETs. Int. J. Distrib. Sens. Netw..

[47] Hu H., Lu R., Zhang Z., Shao J. (2016). REPLACE: A reliable trust-based platoon service recommendation scheme in VANET. IEEE Trans. Veh. Technol..

[48] Amoozadeh M., Raghuramu A., Chuah C., Ghosal D., Zhang H.M., Rowe J., Levitt K. (2015). Security vulnerabilities of connected vehicle streams and their impact on cooperative driving. IEEE Commun. Mag..

[49] Amoozadeh M., Deng H., Chuah C.N., Zhang H.M., Ghosal D. (2015). Platoon management with cooperative adaptive cruise control enabled by VANET. Veh. Commun..

[50] Ren W., Beard R.W. (2008). Overview of consensus algorithms in cooperative control. Distributed Consensus in Multi-vehicle Cooperative Control: Theory and Applications.

